# Small-Angle Twist Grain Boundaries as Sinks for Point Defects

**DOI:** 10.1038/s41598-018-21433-7

**Published:** 2018-02-27

**Authors:** Hao Jiang, Izabela Szlufarska

**Affiliations:** 1 Department of Materials Science and Engineering, University of Wisconsin, Madison, WI 53706 USA; 2 Department of Engineering Physics, University of Wisconsin, Madison, WI 53706 USA

## Abstract

It is known that grain boundaries (GBs) provide sinks for defects induced into a solid by irradiation. At the same time radiation can change the atomic structure and chemistry of GBs, which in turn impacts the ability of GBs to continue absorbing defects. Although a number of studies have been reported for tilt GBs acting as defect sinks, the questions of how twist GBs evolve to absorb non-equilibrium concentrations of defects and whether GBs saturate as defect sinks for typical irradiation conditions have remained largely unanswered. Here, we used a combination of molecular dynamics and grand canonical Monte Carlo simulations to determine how twist GBs accommodate point defects. We used SiC and {001} and {111} twist GBs as model systems. We found that diffusion of defects along GBs in this material is slow and for most experimentally relevant conditions point defects will accumulate at twist GBs, driving structural and chemical evolution of these interfaces. During irradiation, screw dislocations within GB planes absorb interstitials by developing mixed dislocation segments that climb. Formation of mixed dislocations occurs either by nucleation of interstitial loops or by faulting/unfaulting of stacking faults. Both types of twist GBs can accommodate a high density of interstitials without losing the crystalline structure, irrespectively of the interstitial flux.

## Introduction

Exposure to radiation environments leads to formation of point defects and defect clusters in amounts significantly exceeding their equilibrium concentrations. Accumulation of defects can lead to undesirable consequences, such as crystalline-to-amorphous transformation^1^, swelling^2^, and embrittlement^3^, and these phenomena can adversely affect the lifetime of components in nuclear reactors. It is known that interfaces, such as grain boundaries (GBs), can act as sinks for radiation-induced defects^4,5^. Furthermore, the ability of GBs to absorb defects (sink strength) is not constant, but depends on such factors as the radiation environment (e.g., the dose rate, temperature), GB character (i.e., misorientation angles), as well as the evolution of GB’s structure and chemistry during irradiation.

Dependence of GB sink strength on the GB character has been a subject of many recent studies. Both static properties (e.g., formation energies) and dynamic properties (e.g., migration barriers) of defects in pristine GB structures have been investigated^6–12^. For example, Tschopp *et al*.^6^ calculated the distribution of defect binding energy in 170 tilt and twist GBs in Fe and concluded that both the local structure of GBs and the distance to GBs have a significant influence on the magnitude of binding energies. Uberuaga *et al*.^7^ investigated the mobility of both point defects and defect clusters in a few tilt, twist and mixed GBs in Cu. Based on the kinetics of defects in pristine GBs, an object kinetic Monte Carlo model was developed to determine the sink strength of these GBs. The above-mentioned studies have been helpful in understand the interaction between different types of pristine GBs and defects. However, because GB structures can change as defects accumulate at the interface, the energy landscape will evolve correspondingly. For this reason, the knowledge of interactions between pristine GB and defects alone is not sufficient to predict long-term evolution of GBs under irradiation and such long-term evolution is the focus of the present study.

Recently, a few studies have been reported to shed light on this question and most of these studies focus on small-angle tilt GBs^13–18^. It is known that such GBs are composed of sets of edge dislocations, and point defects can be absorbed to edge dislocations by dislocation climb. For example, by loading interstitials to tilt GBs in Mo using molecular dynamics (MD) simulations, Novoselov *et al*.^13^ showed that tilt GBs accommodate defects by climb of edge dislocations and that GB energy evolves in a repeatable pattern as a function of the number of interstitials loaded onto the GB. Similar repeatable patterns in GB energy and free volume were also reported during continuous loading of vacancies into tilt GBs in Cu^14,15^. In addition to dislocation climb, Frolov *et al*.^16^ recently found that the core of edge dislocations in tilt GBs in Cu can reconstruct to different configurations, depending on the number of point defects loaded to the interface. The authors proposed this core reconstruction as one of the possible mechanisms by which tilt GBs can accommodate a certain number of interstitials and vacancies under irradiation. In our earlier study on tilt GBs in SiC^17^, we demonstrated that there is a competition in annihilating defects between dislocation climb and defect diffusion along GBs to other sinks. The relative contributions from each of these mechanisms can be determined using a multiscale model that considers both defect kinetics and a long-term evolution of tilt GB structure^17^.

While interactions between defects and tilt GBs as well as the long-term structural evolution of tilt GBs are relatively well understood, much less is known about how twist GBs evolve to accommodate defects. Compared to edge dislocations in tilt GBs, a network of screw dislocations in twist GBs creates a more complex energy landscape and it is still unclear by what exact mechanism screw dislocations annihilate point defects. So far, only few studies^19,20^ were reported in this area. In 2012, Matínze *et al*.^19^ investigated the segregation of vacancies to {001} twist GBs in Cu using a kinetic Monte Carlo (kMC) model. The {001} twist GBs consist of a square grid of screw dislocations. The authors found that under low vacancy loading rate, vacancies can diffuse to dislocation networks and form voids. Under high vacancy loading rate, vacancies aggregate and form voids both at dislocation cores and in non-dislocation regions within the GB plane. Later on, the same group used a kMC model to investigate segregation of vacancies in {110} and {111} twist GBs^20^. The {110} twist GBs in Fe consist of a network of hexagonal screw dislocations, and the {111} twist GBs in Cu can be described as alternating stacking fault (SF) and unfaulted regions in a triangular shape separated by partial dislocations. A strong preference for vacancies to segregate to dislocation intersections was reported in both cases. Additionally, the authors found that aggregation of vacancies at dislocation intersections in Cu {111} twist GBs can lead to shrinking of the SF area. From these studies, it is obvious that twist GB structure undergoes a noticeable structural change as defects accumulate at the interface. However, the number of vacancies loaded to GBs in these studies is too low (corresponding to 10^−4^ displacement per atom (dpa)) to determine a long-term evolution of twist GBs under irradiation.

Understanding of how twist GBs accommodate defects is important on its own right because such GBs can be present in polycrystalline samples. In addition, studies of twist GBs provide insights into how general GBs (a combination of both tilt and twist components) behave under irradiation, which is still an open question. An interesting aspect related to the behavior of twist GBs under radiation is the question of how screw dislocations absorb defects. Unlike edge dislocations, which climb to absorb point defects in tilt GBs, screw dislocations do not climb. Previous studies found that screw dislocations isolated in a crystal can absorb point defects or Frank loops by forming mixed dislocation in the shape of helical turns^21–25^. These mixed dislocations can climb to absorb defects due to their edge components. However, it is unclear whether a network of screw dislocations in GBs can accommodate defects in a similar way as isolated screw dislocations.

In this study, we investigate defect kinetics in {001} and {111} small-angle twist GBs and elucidate how these GBs evolve to accommodate defects in cubic SiC. Cubic SiC is of interest to applications in the nuclear reactor technologies because of its good mechanical strength, high-temperature stability, and low neutron capture cross-section^26–31^. Here, we focus on behavior of interstitials because defect flux to GBs in irradiated SiC is expected to be dominated by interstitials rather than vacancies based on predictions from *ab initio* based rate theory models^17,32^.

## Results

### Defect segregation and kinetics at {001} twist GBs

**Figure 1:**
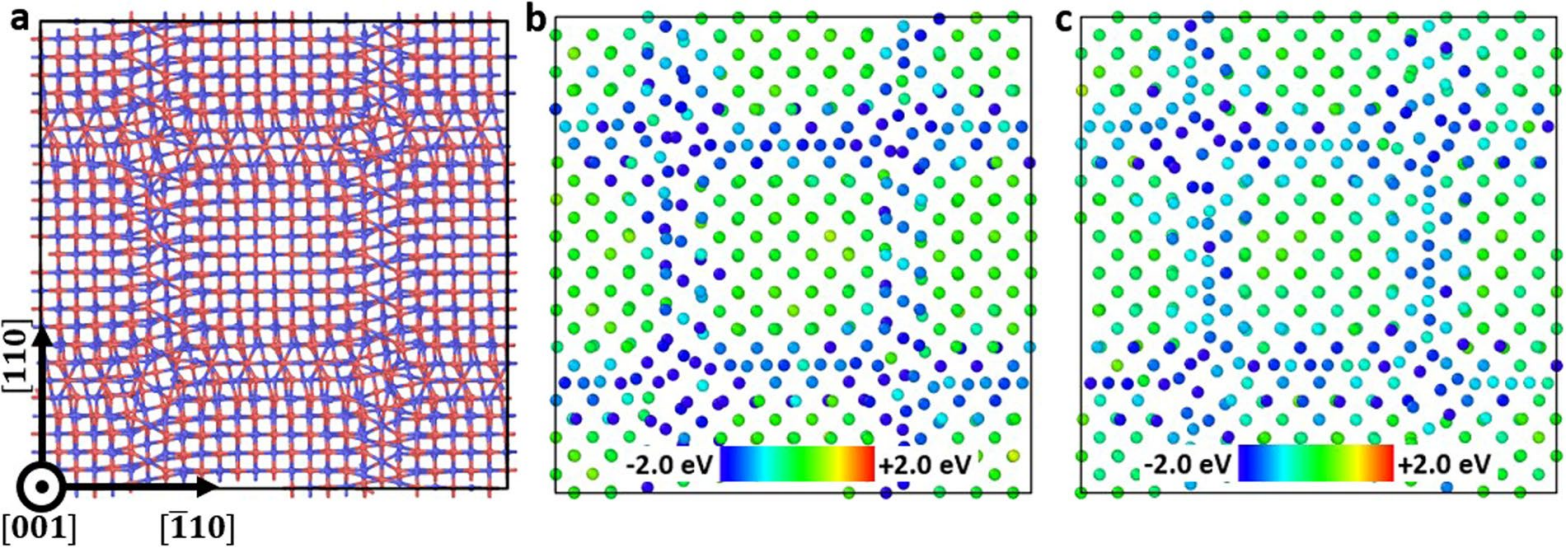
Atomic structure and defect segregation energy in (001) Σ85 *θ* = 8.8° twist GB. (**a**) Atomic structure, where red and blue atoms represent C and Si, respectively; (**b**) Segregation energy of C interstitials; (**c**) Segregation energy of Si interstitials. Segregation energy is defined in equation (1).

In Fig. 1a we show the structure of Σ85 *θ* = 8.8° GB, which is representative of {001} small-angle twist GBs. The interface is comprised of a square grid of screw dislocations with Burgers vectors $\overrightarrow{b}=\frac{a}{2}[110]$ or $\,\frac{a}{2}[\bar{1}10]$, where *a* is the lattice constant of SiC. A similar square grid of screw dislocations within {001} twist GBs have been observed by electron microscopy and atomic simulations in fcc metals, such as Cu^19^ and Au^33^.

With the interface structure determined, we now examine the segregation energy of interstitials at GBs, where segregation energy is defined as

1 $${E}_{seg}={E}_{defect}^{GB}-{E}_{defect}^{bulk}.$$

Here, ${E}_{defect}^{GB}$ is the energy of a bi-crystal supercell with only one interstitial at GB, ${E}_{defect}^{bulk}$ is the energy of the same supercell with only one interstitial in a crystal lattice away from both the GB and the frozen layers (defined in method section). With this definition, a negative value of the segregation energy means it is energy favorable for an interstitial to segregate to that site from a bulk. In the calculations of segregation energy, the interstitial is placed at different sites in the GB, followed by a 50 ps MD simulation in NVT ensemble at 500 K to relax the structure and a fast quenching to bring the system to a local energy minimum. Results are shown in Fig. 1b,c. It is clear that dislocations and dislocation intersections act as strong traps for interstitials at the interface, and segregation to dislocation intersections is stronger than to dislocation lines. More specifically, the segregation of C interstitial is around −2.4 eV on dislocations lines, and approximately −3.4 eV at intersections. The segregation energy of Si interstitial is around −1.8 eV on dislocations, and −3.5 eV at intersections. We have also found that the interaction between defects and GBs is relatively short-ranged. Interstitial formation energies are altered by GBs only within 2 atomic layers (~6 Å) from the GB plane on each side. This is in contrast to tilt GBs where the stress field from edge dislocations extended up to 3 nm away from the GBs^17,34^.

**Figure 2:**
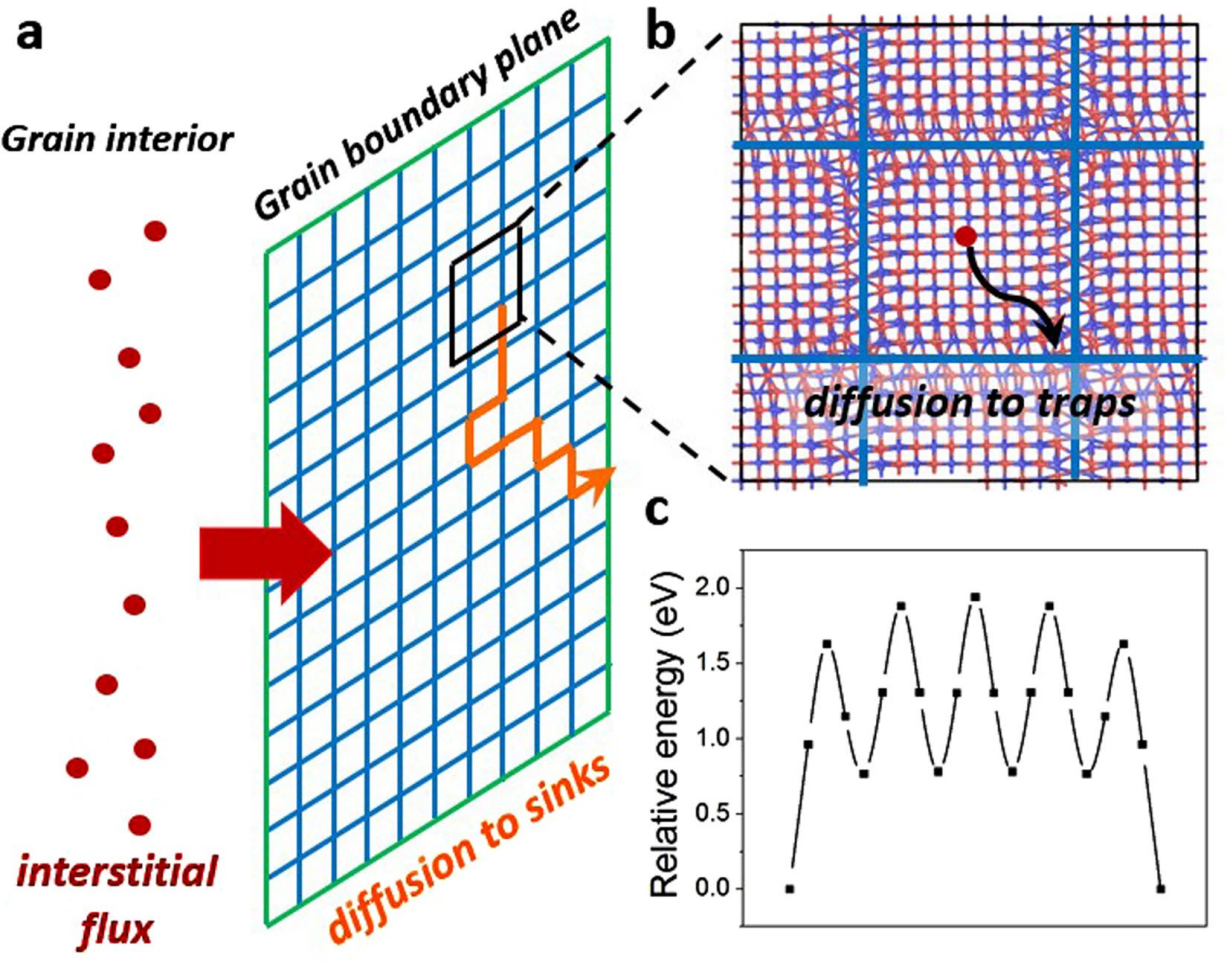
Kinetic processes of interstitials at {001} twist GBs. (**a**) A schematic drawing of the 2D random walk model (explained in detail in text); (**b**) Migration process of an interstitial from a crystalline region in GB to dislocation grid. Blue and red atoms correspond to Si and C, respectively; (**c**) energy landscape for C interstitial migration between dislocation intersections along a screw dislocation.

In our earlier study^17^ we found that tilt GBs play multiple roles in accommodating defects. Depending on the grain size and irradiation conditions, tilt GBs can either act as diffusion channels and transport defects to other sinks (triple junctions or surface), or they can remove defects from the crystalline grains by evolving the GB structure (i.e., by growing new lattice planes through dislocation climb). In order to determine the role of twist GBs in removing defects under various irradiation conditions, here we compare timescale of several kinetic processes in {001} twist GBs. The first process is segregation of interstitials from grain interior to GBs, shown as a red arrow in Fig. 2a. Once interstitials segregated to GBs, they can diffuse within the GB plane through crystalline-like regions to reach GB dislocations (black arrow in Fig. 2b), and then diffuse along dislocation lines on the dislocation grid to reach the ends of GBs (orange trajectory in Fig. 2a). The ends of GBs are assumed to be other defect sinks such as triple junctions or surfaces that can annihilate these defects. The timescales of the above processes determine how fast twist GBs can transport defects to other sinks under a certain defect flux to the interface, and therefore determine whether there will be defect accumulation at twist GBs.

In our analysis we use *t*_*seg*_ to denote the timescale of interstitial segregation to GBs in the units of seconds. Specifically, *t*_*seg*_ is defined as the average time interval between the arrivals of two successive interstitials from grain interior to the GB. *t*_*seg*_ can be calculated from the interstitial flux *J* (#/nm^2^∙s) to GBs, which in turn can be determined for instance from an *ab initio* based rate theory model^32^. Details of implementation and parameters used in this model can be found in ref.^32^. Here, we vary the grain radius *r* of SiC and radiation environments (dpa rate, temperature, total dpa) to consider a wide range of conditions. For simplicity, we assume each GB to have a circular shape with an area of *πr*^2^, and we obtain *t*_*seg*_ = (*J ∙ πr*^2^)^−1^. The time of interstitial migration through crystalline-like region within the GB to dislocations is denoted as *t*_*migr*_ (in the units of seconds), and it can be estimated as

2 $${t}_{migr}=\frac{{(d/2)}^{2}}{{\rm{4}}D}.$$

Here *D* is diffusivity of interstitials in bulk SiC^35^, *d* is the distance between parallel dislocations within the GB, and a factor of 4 is used for 2-dimensional diffusion. We assume the diffusivity of interstitials within crystalline-like regions in GBs to be the same as that in perfect crystal. The time of interstitials diffusion along dislocation grid to reach edges of GBs is denoted as *t*_*sink*_ and it has the units of seconds. To determine *t*_*sink*_, we employ a similar strategy for coarse grained modeling of GBs as implemented in ref^36^. First of all, we use the nudged elastic band (NEB) method^37^ to obtain the energy landscape for interstitials diffusion along a dislocation lines between two intersections of dislocations. The energy landscape for C interstitial diffusion between dislocation intersections is shown in Fig. 2c. This energy landscape was averaged over NEB calculations on 4 different dislocation segments in Fig. 1a. We then perform a one-dimensional kMC simulation to obtain the average time *τ* for one interstitials to hop on this energy landscape from one dislocation intersection to another. Finally, we mesh the GB plane by using nodes to represent intersections and we set the distance between neighboring nodes to be *d*. Simulations of two-dimensional (2D) random walk of interstitials on this GB mesh gives us the average number of steps *N*_*step*_ for interstitials to diffuse from the center of the GB to the edge and we calculate *t*_*sink*_ as *N*_*step*_ × *τ*.

Calculated values of *t*_*seg*_, *t*_*trap*_, and *t*_*sink*_ for SiC in samples with various grain sizes under different irradiation conditions are shown in Table S1 in Supplementary materials. Here we summarize the general trends observed in our simulations. First of all, we find that *t*_*migr*_ ≪*t*_*seg*_ for most grain sizes and irradiation conditions considered in this study. This is because the diffusion distance for interstitials to segregate from crystalline-like regions in GB to dislocations in small angle twist GBs is not larger than a few nanometers. However, the diffusion distance for interstitials to segregate from grain interior to GB is much larger because grain radius can vary from tens of nm to tens of μm. Assuming the same interstitial diffusivity in crystalline regions of the grain and in crystalline-like regions of the GB, it is straightforward to see that *t*_*migr*_ ≪*t*_*seg*._ Based on this analysis we can conclude that one interstitial that segregated to twist GBs can quickly diffuse to dislocations before the arrival of the next interstitial and it is unlikely for two or more interstitials to form clusters in crystalline-like regions within the GB plane. The fact that interstitials can quickly diffuse to dislocations also validates the use of grand canonical Monte Carlo (GCMC) for loading interstitials to GBs. This is because in GCMC, interstitials are most likely to be loaded to sites with low formation energies (i.e., dislocations and intersections), instead of crystalline regions within the GB. The second trend that emerged from our calculations is that *t*_*seg*_ ≪*t*_*sink*_ for most conditions considered in this study. This finding implies that it takes much longer for one interstitial to diffuse along the dislocation grid in GB to reach sinks than to segregate to GBs from the grain interior. The underlying reason is that the energy barrier for an interstitial to be released from dislocation intersections is relatively high (e.g., 1.6 eV for C interstitial as shown in Fig. 2a) as compared to diffusion barriers (0.74 eV in bulk^38^). We can therefore conclude that for most cases interstitials will accumulate at dislocation intersections in twist GBs and will likely drive GB structural change. An exception to this trend is the case of SiC with a small grain size (below 100 nm), irradiated at low dose rate (below 10^−5^ dpa/s), and at a high temperature (over 873 K). Under these conditions, interstitials can frequently escape from dislocation intersections and they can diffuse to sinks faster than they accumulate within the GB plane. A similar analysis of defect kinetics can also be done for Si interstitials. Si interstitial on a dislocation line has a higher energy than Si interstitial at a dislocation intersection by about 1.7 eV, which means that the energy of at least 1.7 eV has to be provided to Si interstitial to become untrapped from the intersection. This barrier is already higher than the corresponding energy of 1.6 eV for C interstitials and therefore the conclusion that interstitials can accumulate at twist GBs is also valid for Si interstitials. Therefore, one can conclude that interstitials will accumulate on the dislocation grid (especially at dislocation intersections), in {001} twist GBs under most irradiation conditions.

### Evolution of the atomic structure of {001} twist GBs under interstitial flux

**Figure 3:**
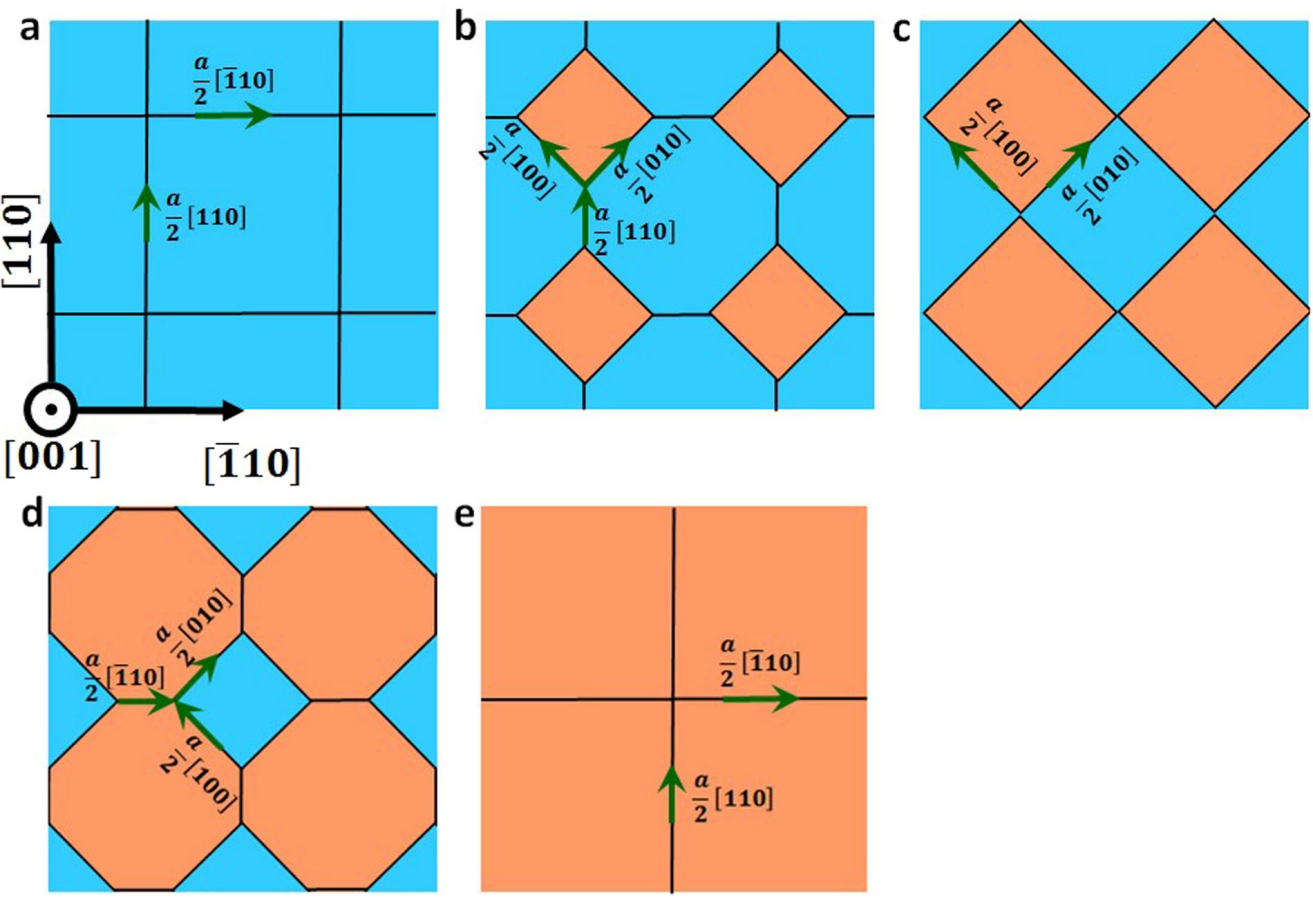
Schematic representation of loop nucleation and extension within {001} twist GBs under interstitial flux. Black lines represent dislocations, and green arrows represent the screw components of the Burgers vector of each dislocation segment.

Since we have shown that interstitials accumulate in {001} twist GBs, we now need to determine how GBs evolve to accommodate these defects. To do so, we employ a hybrid MD and GCMC technique to load a stoichiometric flux of C/Si interstitials onto Σ85 twist GB. We find that {001} twist GBs accommodate interstitials by nucleation and extension of interstitial loops. This process is schematically shown in Fig. 3 and the actual snapshots from simulation trajectories are shown in Supplementary materials. Starting from a pristine GB (Fig. 3a), the first set of interstitials becomes trapped at dislocation intersections because of the low segregation energy at these sites. As interstitials accumulate at dislocation intersections, they reorganize to nucleate dislocation loops in a square shape (see Fig. 3b). The edges of the loop consist of mixed dislocations with both edge and screw component. In the example of (001) twist GB in Fig. 3, the Burgers vector of the edge component is ${\overrightarrow{b}}_{E}=\frac{a}{2}[001]$ and the Burgers vector of the screw component is either ${\overrightarrow{b}}_{S}=\frac{a}{2}[010]$ or $\,\frac{a}{2}[100]$. The edge component of ${\overrightarrow{b}}_{E}=\frac{a}{2}[001]$ describes the lattice mismatch induced by an interstitial loop lying in the (001) plane. The screw components (green arrows in Fig. 3) are necessary to accommodate the relative twist between the top and the bottom SiC crystals. With more and more interstitials attached to interstitial loops, these loops extend (Fig. 3c) and connect with each other (Fig. 3d) to form pure screw dislocations between the loops. The Burgers vector of these pure screw dislocations are $\overrightarrow{b}=\,\frac{a}{2}[110]$ or $\frac{a}{2}[\bar{1}10]$, which are the same as those in pristine GB (Fig. 3a). These interstitial loops continue to extend by absorbing interstitials until a complete (001) plane is grown (Fig. 3e). If we continue loading additional interstitials into the GB structure shown in Fig. 3e, these interstitials can nucleate loops at dislocation intersections again, where the shape of the loops is the same as the blue regions shown in Fig. 3d. These loops then extend (see the change in the shape and size of blue regions from Fig. 3d to Fig. 3c,b) to grow another (001) plane (Fig. 3a).

**Figure 4:**
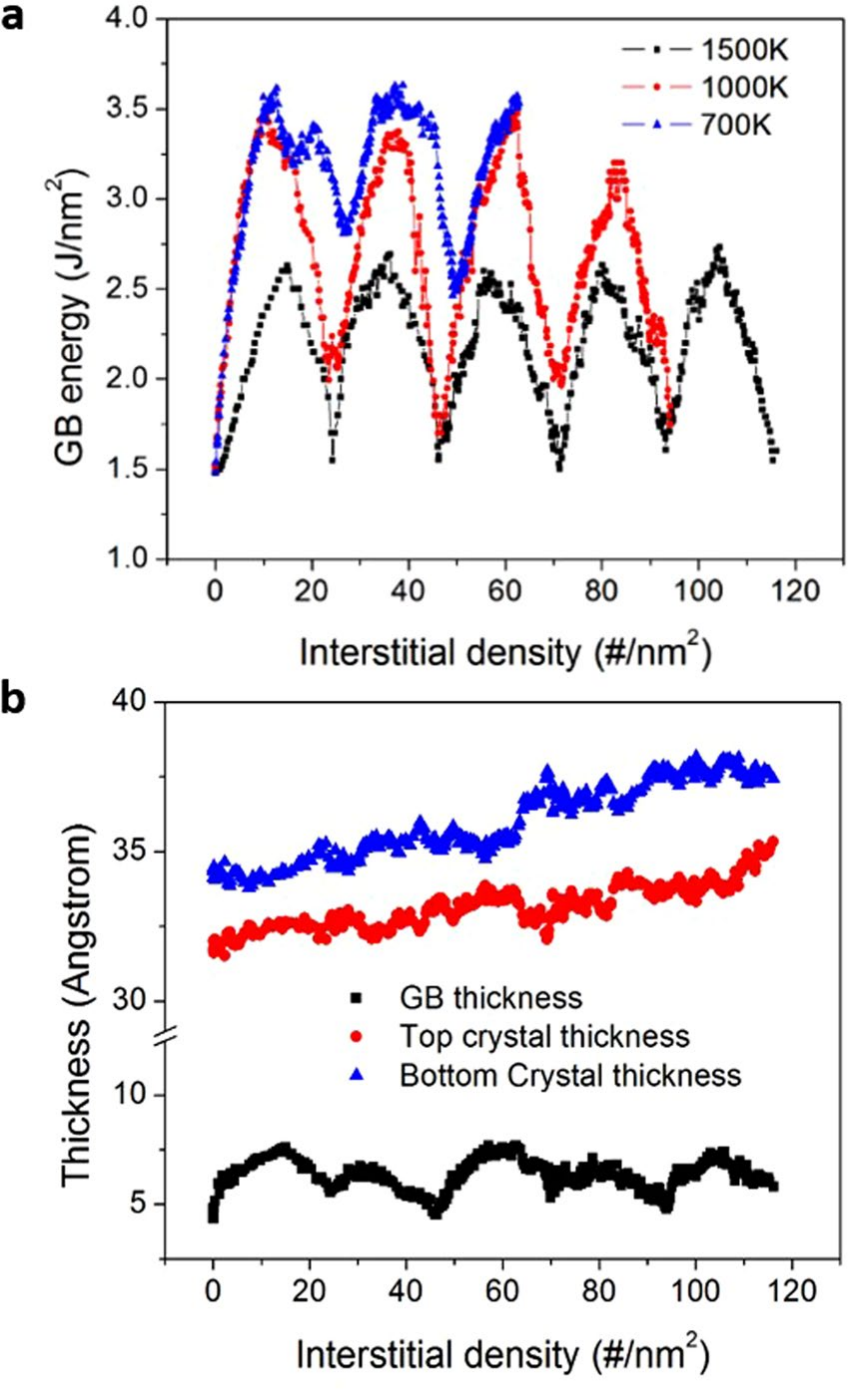
Repeating patterns in the evolution of {001} twist GB under interstitial flux. (**a**) GB energy; (**b**) Thicknesses of the GB, the top, and the bottom crystal.

The pattern of absorbing interstitials by loop nucleation and extension and continuous growth of (001) planes can also be characterized in terms of GB energy and GB thickness, shown in Fig. 4. GB thickness is calculated as twice the standard deviation from *z* coordinates (z axis is perpendicular to GB plane) of atoms in dislocation core, as identified by the structural analysis function implemented in Ovito^39^. As one can see from Figs. 4a,b, GB energy and GB thickness exhibit a periodic pattern as a function of the density of loaded interstitials. Because crystal layers grow from loop nucleation, the thicknesses of the top and the bottom crystals grow approximately linearly with the density of interstitials. This pattern implies that {001} twist GBs do not easily saturate in their ability to absorb interstitials.

Loop nucleation and extension are kinetic processes that depend on both the loading rate of interstitials and the temperature at which the supercell is annealed. Since in simulations it is not possible to explore a wide range of loading rates, we perform simulations at various temperatures. Qualitatively, trends observed at a higher temperature are representative of trends that would be observed at a lower loading rate. The effect of temperature on the trend in GB energy with the interstitial density is shown in Fig. 4a. At lower temperatures, the maxima and minima in the GB energy are generally shifted to higher energies as compared to those relaxed at higher temperatures. This is because at lower temperatures slow kinetics cannot fully optimize the structure within the limited timescale of MD simulations. However, the repeatable pattern is clear at temperature as low as 700 K, even on MD timescales. This result implies that loop nucleation and extension induced by interstitial flux to {001} twist GBs is generally expected over a wide range of temperatures, even more so at slower loading rates typically encountered in experiments. We also examined how the stoichiometry of the interstitial flux affects the evolution of the GB. This is because the defect flux in SiC is usually assumed to be C-rich as predicted from rate theory models^32^, but the exact C/Si ratio is unknown. Here, we vary the stoichiometry of interstitial flux from stoichiometric (C:Si = 1:1) to C only interstitial flux. We found that the cyclic pattern in GB energy occurs for a wide range of stoichiometries. In the case where the flux is C-rich, C is accommodated at GBs by forming C antisites, i.e., C atoms occupying Si sublattice.

### Defect segregation and kinetics at {111} twist GBs

**Figure 5:**
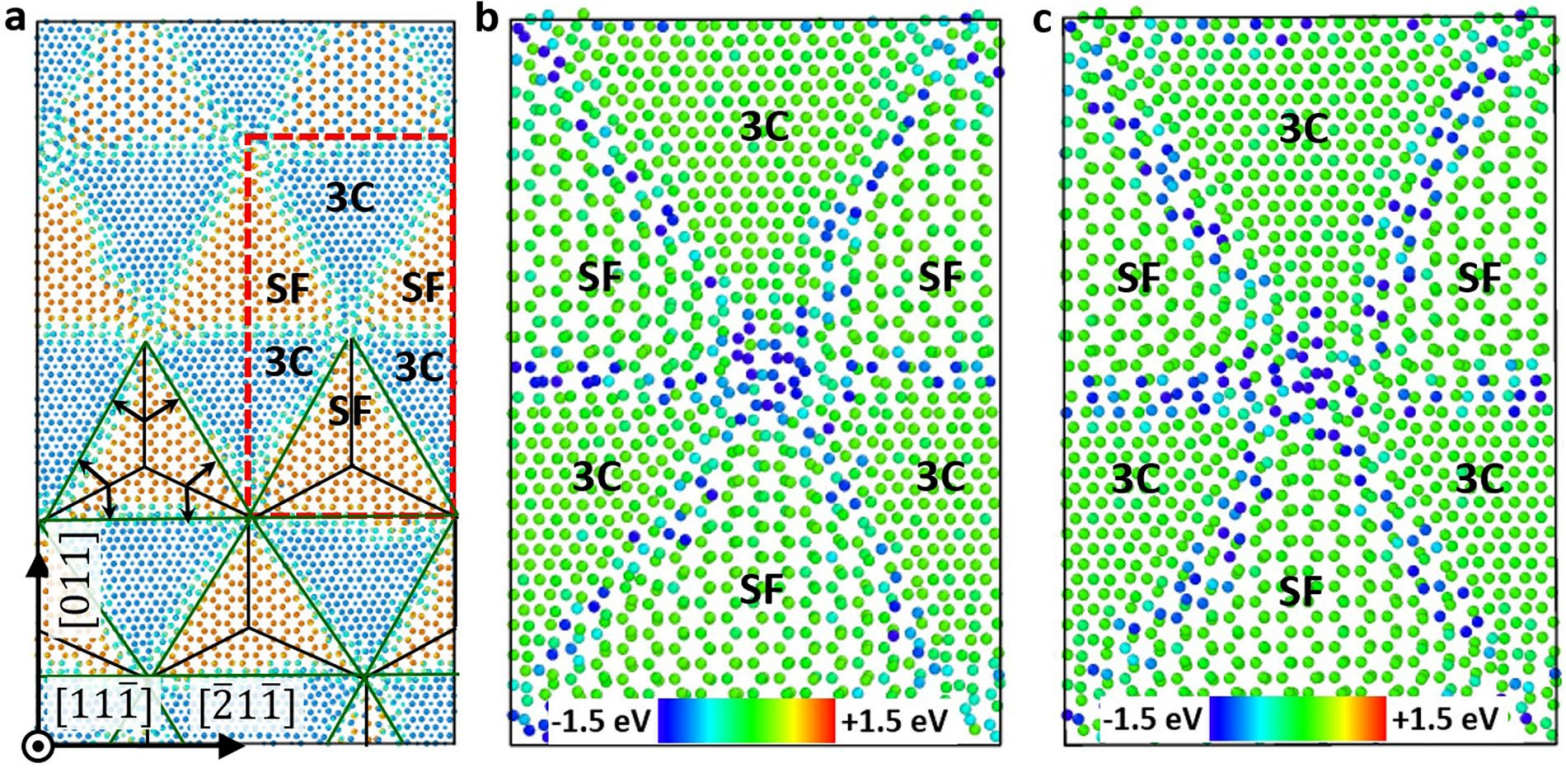
Atomic structure and defect segregation energy at $(11\bar{1})$ Σ507 *θ* = 4.4° twist GBs. (**a**) GB structure. (**b**) C interstitial segregation energy; (**c**) Si interstitial segregation energy. In (**a**) atoms in blue have cubic SiC (3C) structure, atoms in red are in a stacking fault (SF), and atoms in cyan belong to partial dislocations. Thin black lines represent the $\frac{a}{2}\langle 110\rangle$ screw dislocations before faulting, thin green lines represent the $\frac{a}{6}\langle 211\rangle$ partials after faulting. The black arrows show the formation of stacking faults by dissociation of perfect screw dislocations into partial dislocations. The red dashed square shows the region where interstitial segregation energies are calculated and shown in panels b and c.

The common structure of {111} small-angle twist GB is shown in Fig. 5a using $(11\bar{1})$ Σ507 *θ* = 4.4° as an example. The GB plane is composed of alternating triangular stacking fault (SF) regions and cubic regions (3C). The SF regions are formed due to the dissociation of perfect screw dislocations (thin black lines in Fig. 5a) into partials (thin green lines in the same figure) according to the following reaction

3 $$\frac{a}{2}[\bar{1}10]\to \frac{a}{6}[\bar{1}21]\,+\frac{a}{6}[\bar{2}1\bar{1}].$$

The SF regions are intrinsic stacking faults with stacking sequence of (ABC)(BC)(ABC) along the [111] direction. Similar structure has been reported from experimental observations and simulations of fcc metals with low SF energy, such as Cu^40,41^. The SF energy in 3C-SiC is negative^42,43^, which means it is energetically favorable to form SF and partial dislocations.

With the most stable structure identified, we now examine the segregation energy of interstitials at different sites in these GBs. The results for Σ507 GB are shown in Fig. 5b,c for C and Si interstitials, respectively. It is clear that partial dislocations and dislocation intersections provide strong traps for interstitials and, additionally, segregation energies at dislocation intersections are lower than those at partial dislocations. More specifically, segregation energy of C interstitials is zero in 3C region, approximately −0.27 eV in the SF region, between −1.4 eV and −1.0 eV on partial dislocations, and between −1.9 eV and −1.3 eV at dislocation intersections. Segregation energy of Si interstitials is zero in both 3C and SF regions, between −2.0 eV and −1.2 eV on partial dislocations, and between −2.1 eV and −3.1 eV at dislocation intersection. The strong attraction of C and Si interstitials to dislocation intersections in the {111} GB is reminiscent of a similar trend that we found in the case of {001} twist GBs.

Here we perform a similar analysis of *t*_*seg*_, *t*_*migr*_, and *t*_*sink*_ to that conducted for {001} twist GBs in order to investigate defect accumulation in {111} twist GBs. First, the time interval *t*_*seg*_ between the arrivals of two successive interstitials from grain interior to the GB is calculated based on the interstitial flux as predicted by *ab initio* based rate theory model^32^. The time *t*_*migr*_ of interstitials migration within crystalline-line region inside the GB to dislocations is calculated by equation (2) based on the distance between parallel partials in {111} twist GBs. The time *t*_*sink*_ of interstitials diffusion along the dislocation grid within GB to edges of GBs is calculated by using a two-dimensional random walk model on a triangular mesh, which preserves the major structural features of {111} twist GB, as shown in Fig. 5a. The nodes of each triangle represent dislocation intersections and the lines of each triangle represent partial dislocations. The time *τ* for an interstitial to move from one dislocation intersection to a neighboring one is estimated based on the energy landscape for migration of an interstitial along a partial dislocation connecting the two intersections (Supplementary materials). The number of hops *N*_*step*_ between dislocation intersections for interstitials to diffuse to sinks can be calculated by the two-dimensional random walk model and the *t*_*sink*_ can be calculated as *N*_*step*_ × *τ*. Values of *t*_*seg*_, *t*_*trap*_, and *t*_*sink*_ under various irradiation conditions are reported in Supplementary materials. Similarly as in the {001} twist GBs, interstitials are found to quickly diffuse to partials because *t*_*trap*_ ≪*t*_*seg*_. At low temperature (below 873 K) and high irradiation dose rate (higher than 10^−4^ dpa/s) in SiC with large grains (diameter > 100 nm), we found that the time *t*_*sink*_ for interstitials to diffuse along GB to reach other sinks is much longer than time interval *t*_*seg*_ between arrivals of two successive interstitials from grain interior to the GB. In such scenario, interstitials are likely to accumulate in the GB plane, which in turn raises the question of how the atomic structure of the GB evolves due to interstitial loading. This structural evolution will be discussed in detail in the next section. In SiC with small grain sizes (grain diameter < 100 nm), irradiated at high temperature (over 873 K), and at a relatively low dose rate (lower than 10^−4^ dpa/s), *t*_*sink*_ is comparable or even smaller than *t*_*seg*_. In these cases, defects segregated to GBs can quickly diffuse along the dislocation grid to reach other sinks.

### Evolution of the atomic structure of {111} twist GBs under interstitial flux

**Figure 6:**
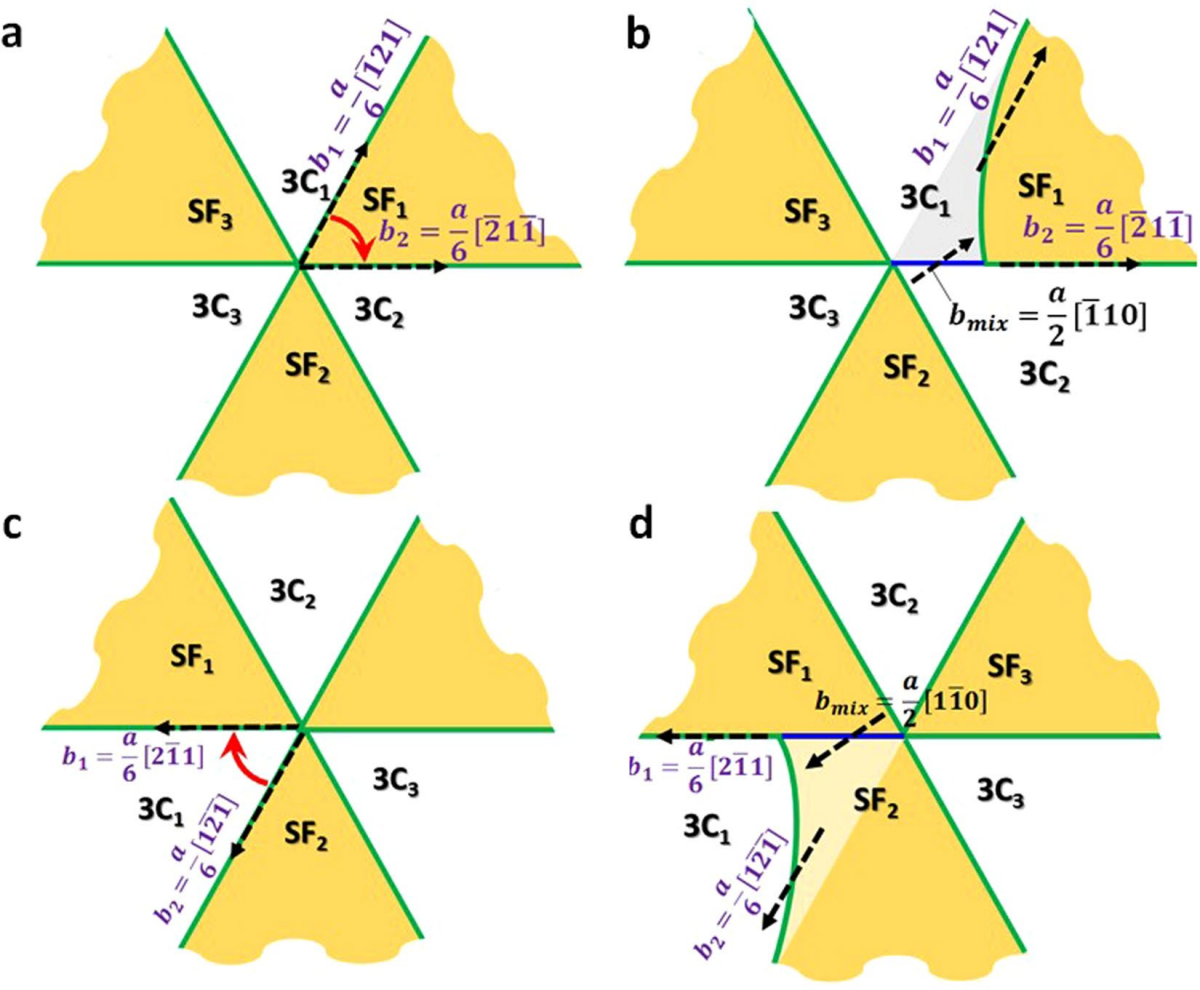
Formation of mixed dislocations in a $(11\bar{1})$ Σ507 *θ* = 4.4° twist GB . (**a**) before and (**b**) after unfaulting; (**c**) before and (**d**) after faulting process. Green lines are partial dislocations, and blue lines are the newly formed mixed dislocations. Black dashed arrows show the direction of the Burgers vector of each dislocation segment. Red arrows in panels a and c show the glide direction of one partial dislocation towards another partial dislocation.

To explore how {111} twist GBs evolve to accommodate interstitials defects, we employ the hybrid MD/GCMC simulation to load GBs with interstitials in different stoichiometry (i.e., different C/Si ratio). By investigating the response of {111} twist GBs with different twist angles from 4.0° to 13.2°, we found that these GBs absorb interstitials by the climb of mixed dislocations. In the following text, we use a representative example of $(11\bar{1})$ Σ507 *θ* = 4.4° twist GB loaded with only C interstitials shown in Fig. 6 to illustrate the formation and climb of mixed dislocations. The mixed dislocations are formed either through a shrinkage of SFs (unfaulting, shown in Fig. 6a,b) or by an extension of SFs (faulting, shown in Fig. 6c,d). During the unfaulting process, one partial dislocation glides towards a neighboring partial dislocation that is separated by a SF (Fig. 6a). This process produces a mixed dislocation according to the following reaction

4 $$\frac{a}{6}[\bar{1}21]\,+\frac{a}{6}[\bar{2}1\bar{1}]\to \,\frac{a}{2}[\bar{1}10]$$

The mixed dislocation has an edge component of ${\overrightarrow{b}}_{E}=\frac{a}{4}[011]$ and screw component of ${\overrightarrow{b}}_{S}=\frac{a}{4}[\bar{2}1\bar{1}]$. The edge component of the mixed dislocation can absorb interstitials by climbing along the $[11\bar{1}]$ direction. During the faulting process, one partial dislocation glides towards a neighboring partial dislocation that is separated by a crystalline-like (3C) region (Fig. 6c). Similarly as in the unfaulting process, faulting produces a mixed dislocation that can climb along the $[11\bar{1}]$ direction. It is important to note that the faulting and unfaulting processes are energy favorable only when interstitials are present at the GB. This is because the newly formed mixed dislocation can climb to absorb interstitials so the system energy can be lowered by moving interstitials to grow a crystal lattice. However, because perfect dislocations have a higher elastic energy than dissociated partials in SiC, these processes are not energetically favorable without the presence of interstitials.

**Figure 7:**
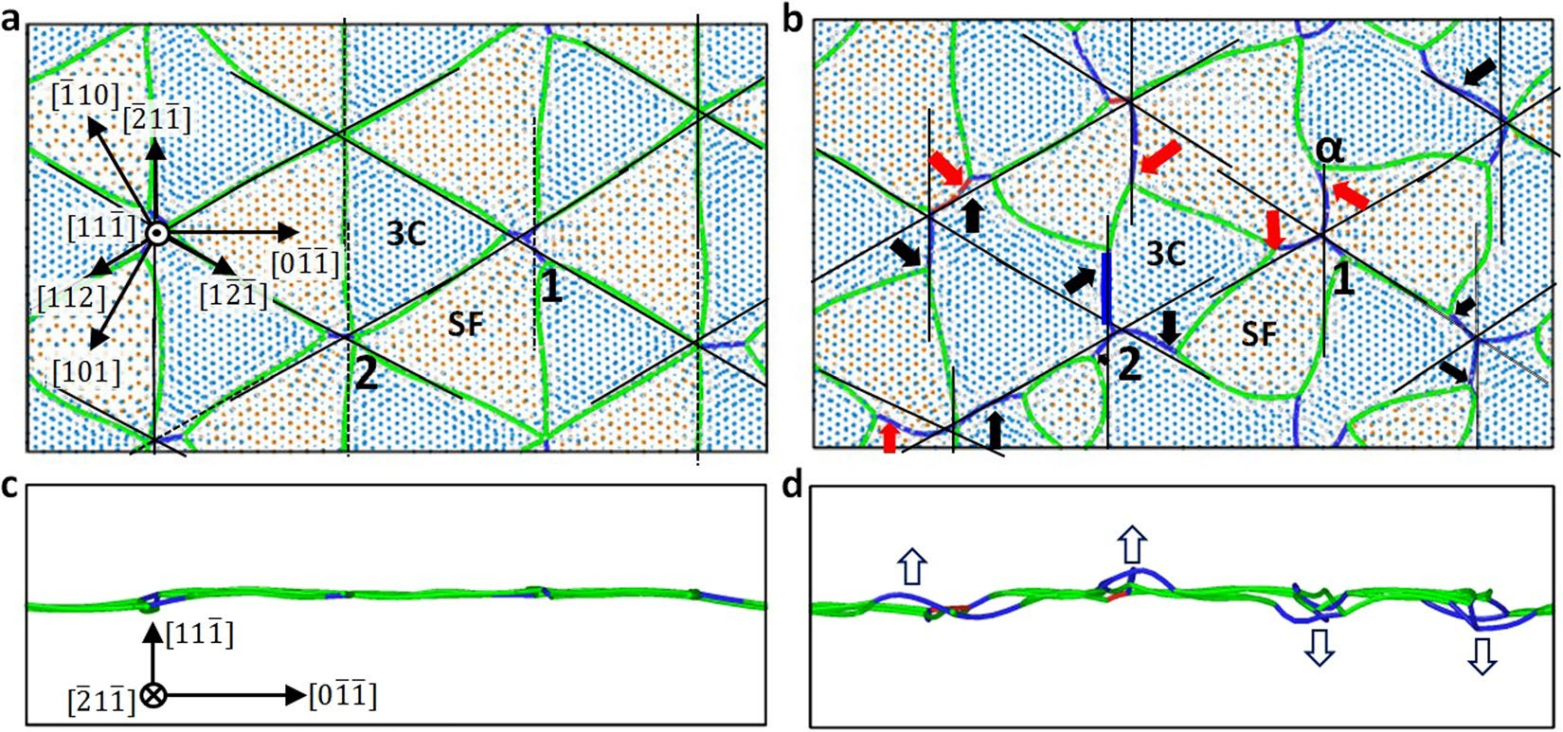
Evidence of faulting and unfaulting processes in a $(11\bar{1})$ twist GB induced by interstitial loading. (**a**) Pristine GB without any interstitials; (**b**) GB loaded with 215 C interstitials; (**c** and **d**), in-plane view of GBs in panels (a and b), respectively. Green lines are partial dislocations with Burgers vectors $\frac{a}{6}\langle 211\rangle$ and blue lines are perfect dislocations with Burgers vectors $\frac{a}{2}\langle 011\rangle$. Thin black lines serve as a reference to note the crystal direction defined with respect to the 3C regions shared by the two crystals. The red and black arrows in panel (b) show the faulting and unfaulting processes that lead to formation of mixed dislocations as detailed in Fig. 6. The thick blue line in panel (b) marks the mixed dislocation segment whose atomic structure will be described in detail in Fig. 8. The arrows in panel (d) show the climbing directions of mixed dislocations. Dislocations are identified and visualized by dislocation analysis implemented in Ovito^46^.

**Figure 8:**
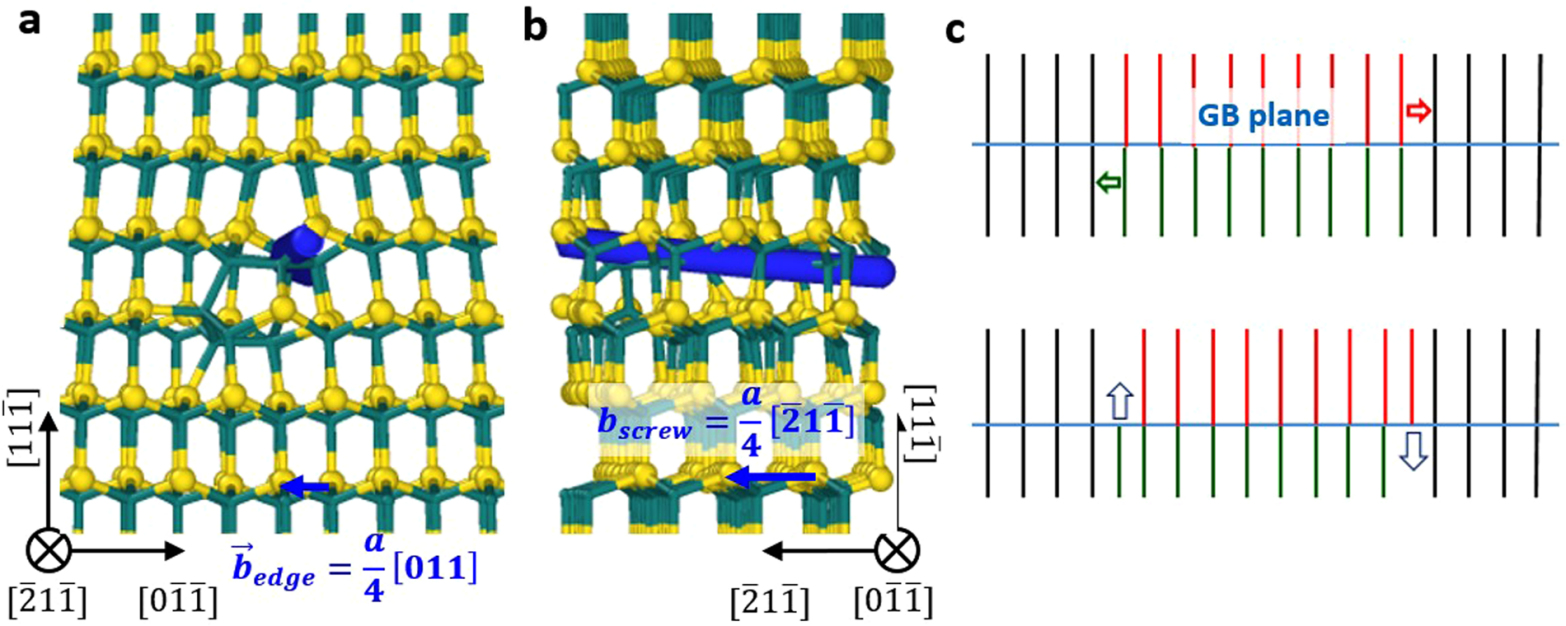
Atomic structure around a dislocation segment highlighted by a thick blue line in Fig. 7b. (**a**) Projection along the dislocation line direction; (**b**) Projection perpendicular to the dislocation line; (**c**) Formation of a dislocation dipole by local shifting of atom planes. In panel (a and b), large yellow spheres are Si, small green spheres are C, the blue line is added to mark the dislocation line, and the blue arrow is the Burgers vector determined from a Burgers circuit.

Evidence for formation and climb of mixed dislocations is shown in Figs 7 and 8. A network of partial dislocations in a pristine GB is shown by green lines in Fig. 7a, and in-plane view of the GB is shown in Fig. 7c. The structure of GBs loaded with 215 C interstitials (corresponding to the density of 1.93/nm^2^) and in-plane view of the GB are shown in Fig. 7b,d, respectively. It is clear that the GB structure changes significantly due to the loading of interstitials, and this change can be explained by the formation and climb of mixed dislocations. One example is the dislocation intersection labeled as “1” in Fig. 8a,b where initial 3C regions around it were faulted to become SF (this process is shown by red arrows in Fig. 7b). The newly formed dislocations (blue lines in Fig. 8b) align along the <211> directions within the interface but not with their Burgers vectors of <011>. The mismatch between dislocation line direction and Burgers vector implies these dislocations have mixed character. Another example is the dislocation intersection labeled as “2” where all initial SF regions around it were unfaulted to become 3C regions (this process is shown by black arrows in Fig. 7b). The detailed atomic structure around the mixed dislocation segment highlighted by the thick blue line in Fig. 7b is shown in Fig. 8a,b. This mixed dislocation is formed by the reaction described by equation (4). From the projection along the dislocation line direction in Fig. 8a, we can identify Burgers vector of the edge component of the dislocation, which is $\frac{a}{4}[011]$ (blue arrow). This vector is determined by drawing a Burgers circuit around the dislocation core. Similarly, from the projection perpendicular to the dislocation line, we can identify the vector for the screw component of the dislocation, which is $\frac{a}{4}[\bar{2}1\bar{1}]$ (blue arrow).

The newly formed mixed dislocations (blue segments in Fig. 7b) climb out of the GB plane to accommodate loaded interstitials, as labeled by the arrows in Fig. 7d. One should note that some mixed dislocations climb along the $[11\bar{1}]$ direction while others climb in the opposite direction which is $[\bar{1}\bar{1}1]$. This is because there is an extra half plane for each mixed dislocation when looking along the dislocation line direction (e.g., Fig. 8a). However, the total number of atomic planes in the supercell is conserved. Therefore, the extra half plane has to be compensated at another location by a mixed dislocation with an opposite edge component vector and with an extra half plane on the other side of the GB. This process resembles the formation of an edge dislocation dipole by shifting atomic planes between two edge dislocations, as shown in Fig. 8c. Mixed dislocations within a dipole have the opposite edge component vectors and climb in the opposite directions. Another feature of the mixed dislocation is that they are pinned to partial dislocations in the GB plane. To be more specific, the end of one mixed dislocation line connected to two partial dislocations (e.g., point α in Fig. 7b) is pinned to the partial dislocations that reside within the GB plane. The other end of the mixed dislocation, which is connected with other mixed dislocations (e.g., dislocation intersection labeled as “1” in Fig. 7b), can move out of the GB plane if these mixed dislocations climb. The fact that one end of the mixed dislocation is pinned while the other end is free to move by climb is the reason why the mixed dislocations lines develop curvatures in the in-plane view (Fig. 7d).

**Figure 9:**
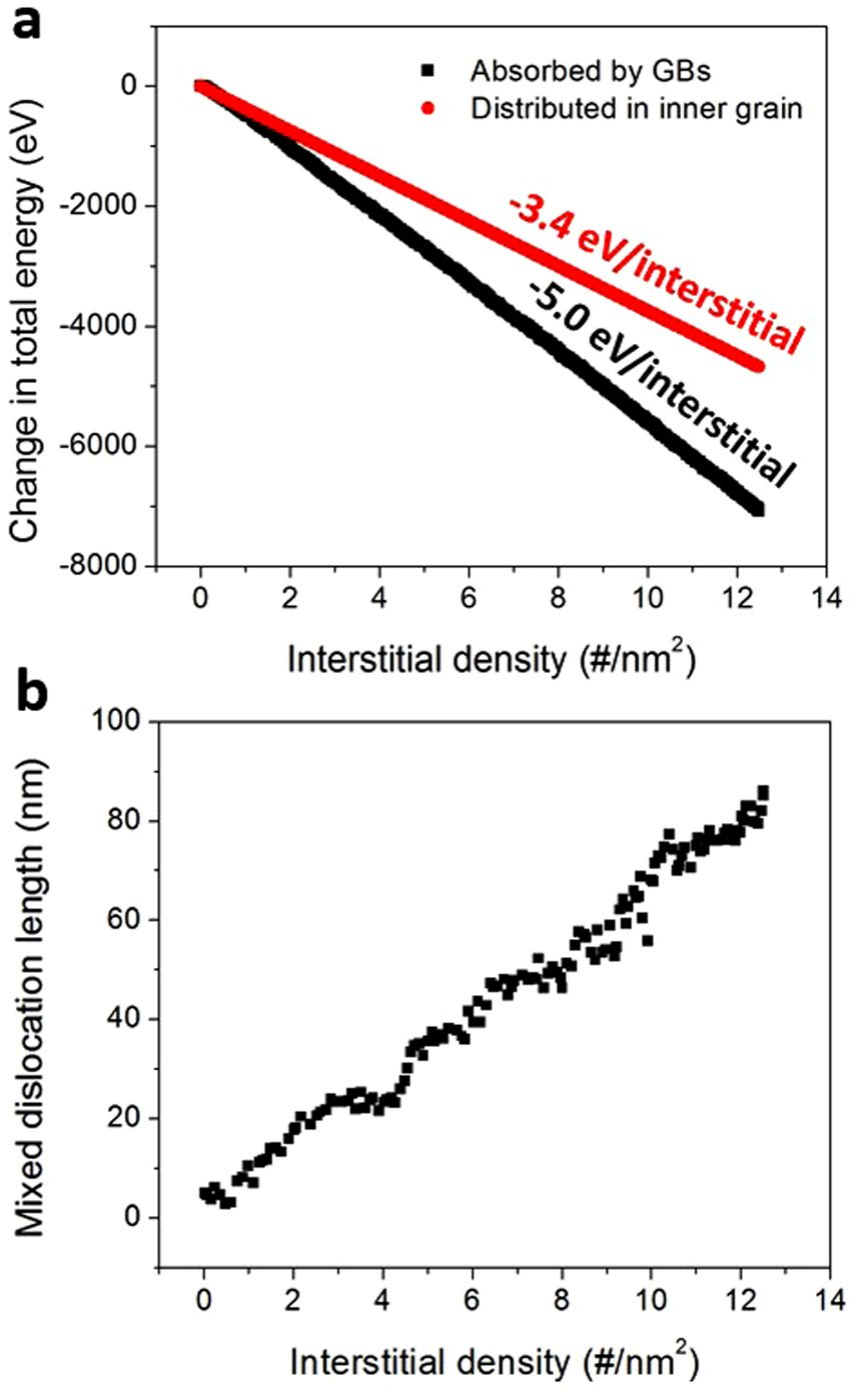
Long term evolution of {111} twist GBs under C interstitial flux. (**a**) change in the supercell energy with an increasing number of interstitials in a GB (black) and in a crystalline grain (red); (**b**) length of mixed dislocations.

The long term evolution of {111} twist GBs induced by absorbing interstitials is quantified in Fig. 9. In Fig. 9a, we plot the change in bi-crystal supercell energy as a function of interstitial density for two cases. The black points represent the change in energy of supercell quenched to 0 K using conjugate gradient from snapshots of C interstitial loading simulation. The red points are calculated as *N* × Δ*E*_*c*_, where *N* is the number of C interstitials and Δ*E*_*C*_ is the average change in energy induced by a single C interstitial in bulk SiC (as reported in ref.^38^ for the same empirical potential). Therefore, the red points are representative of the change in energy of the same bi-crystal supercell, which contains C interstitials in crystalline grains away from GB. By comparing the trends of black and red points in Fig. 9a, it is evident that segregation of interstitials to GBs is energetically favorable even after the GB has been already loaded with multiple interstitials. Figure 9b shows the relationship between the density of loaded interstitials and the length of mixed dislocations. The approximately linear trend is consistent with our previous observations shown in Figs 7 and 8 that the formation and climb of mixed dislocations accommodates loaded interstitials. No sign of amorphization is observed even at the interstitial concentration as high as 12.5 interstitial/nm^2^. We have also varied the stoichiometry of C/Si atoms loaded onto the GB and we found similar trends to those shown in Figs 7–9 for C:Si = 1:1 loading. The main difference is that in the case of an off-stoichiometric interstitial flux, antisites can accumulate at the GB.

## Discussions and Conclusions

We found that interstitial intersections play an important role in determining interstitial kinetics and in accommodating interstitials in twist GBs. This is because dislocation intersections are strong traps for interstitials, and therefore they retard the mobility of interstitials along the dislocation grid within the GB. In addition, the trapping effect causes interstitial aggregation at dislocation intersections and the structural evolution of GBs is therefore initiated at intersections.

We have also found that although both pristine small angle {001} and {111} twist GBs consist of only screw dislocations, they absorb interstitials by developing mixed dislocation segments that can climb. However, unlike isolated screw dislocations that form mixed dislocations in the shape of helical turns^21–25^, interconnected screw dislocations in twist GBs vary in the way of forming mixed dislocations. In {001} twist GBs, interstitial loops nucleate at dislocation intersections in the GB plane and mixed dislocations are formed on the periphery of interstitial loops. These mixed dislocations climb to absorb interstitials and lead to the extension of the interstitial loops in the GB plane. In {111} twist GBs, one partial dislocation glides toward another partial dislocation to form a mixed dislocation. This process can either fault or unfault the region between the two partials to produce or remove SF, respectively. The mixed dislocation can climb out of the GB plane to absorb interstitials. A similar behavior of {111} twist GB was reported in Cu^20^. Upon loading a small number of vacancies, the authors found that vacancies segregated to dislocation intersections and caused the shrinkage of neighboring SFs. However, the number of vacancies loaded at GBs was too low (corresponding to 10^−4^ dpa) to observe significant structural change and therefore the long-scale evolution of twist GBs could not be inferred from this study. Here, by loading a high concentration of interstitials into GBs, we provide an in-depth analysis of GB structural evolution in terms of dislocation reactions and we demonstrate the long timescale evolution of GBs upon continuous defect loading.

In the end, we conclude that small angle {001} and {111} twist GBs can accommodate a large number of interstitials by a structural evolution. In {001} twist GBs, the continuous nucleation and extension of interstitial loops can grow lattice planes at the interface and therefore {001} twist GBs are not easily saturated by interstitials. In {111} twist GBs, interstitials are also converted to lattice planes by climb of mixed dislocations and crystal structures are preserved even for a high density of interstitials loaded into GBs. However, one should keep in mind that the dislocation climb may not continue to absorb an infinite number of interstitials at GBs because of other competing processes. For instance, in a polycrystalline sample strain can build up as more and more lattice planes grow at GBs and the accumulated strain field may affect the rate of defect segregation to GBs or the ways in which they are accommodated at GBs. In SiC, as interstitials segregate to GBs due to their fast mobility, a high concentration of vacancies can build up in grain interior. The high concentration of vacancies may lead to lattice collapse and amorphization^44^. Finally, if there is a high concentration of vacancies near the GB, one may also need to consider emission of interstitials from GBs^4^ and their recombination with vacancies. Therefore, in order to fully understand the irradiation response of poly-crystalline materials, one has to consider coupling of all defect-related processes, including the evolution of GBs as well as of the crystalline grains.

## Methods

{001} and {111} twist GBs with twist angles between 4° to 13° were constructed in bi-crystal cells, as shown in Fig. 1 in Supplementary materials. Two crystals are twisted with respect to each other and stacked along the *z* axis. The GB is initially placed at *z* = 0. The dimensions along the *x* and the *y* axes are determined by the twist angle because the minimum cell size that can satisfy periodic boundary condition (PBC) varies from case to case. The top four atomic layers in the top crystal and the bottom four layers in the bottom crystal are treated as blocks that move freely along the z axis, but the atoms inside the block are not allowed to relax. The presence of the two blocks ensures that the twist angle between the two crystals does not change during thermal annealing. Atoms inside the blocks occupy sites corresponding to a perfect crystal lattice, so this boundary condition effectively represents a semi-infinite grain on either side. The top and bottom crystals are at least 2 nm thick to avoid interactions between the GB and the two blocks. Because we apply PBC in all three directions, a vacuum layer is added to each block to avoid unphysical interactions between the two blocks. All GBs are relaxed in the constant pressure – constant temperature (NPT) ensemble with zero external pressure and at 2500 K for 20 ns, followed by a quenching process to 0 K within 1 ns. The Gao-Weber potential^38^ is used in our MD simulations because it has been shown to correctly describe GB structures^17^ as well as to give point defect properties in good agreement with *ab initio* calculations^35,45^. After MD relaxation, we found similar atomic structures within each group of twist GBs, and therefore here we will use (001) Σ85 *θ* = 8.8° and (111) Σ507 *θ* = 4.4° as representative examples of these boundaries. Details on twist angles and GB energies of all the GBs investigated in this study are given in Supplementary materials.

Loading of defects into the GBs is accomplished using a hybrid grand canonical Monte Carlo (GCMC) and MD technique as implemented in LAMMPS. In GCMC, a C or a Si atom is first inserted at a random position in a predefined zone (this zone will be defined later), then the energy of the inserted atom in that position is calculated and compared with a predefined external chemical potential *μ*. The lower the energy of the inserted atom with respect to *μ*, the higher the chance this particular insertion event will be accepted. Therefore, GCMC ensures that atoms are more likely to be inserted into energy favorable sites. This feature makes GCMC an excellent tool for exploring long term evolution of GBs due to defect accumulation if we assume defects can have enough time to diffuse to low energy sites in the GB plane (this assumption is validated in result section). The hybrid GCMC/MD technique ensures that the structure is able to relax between interstitial insertions performed with GCMC and is carried out as follows. We perform constant volume – constant temperature (NVT) MD simulations, and after every *N* MD time steps the GCMC code is called *M* times to load interstitials. The loading zone for GCMC is set as a 1 nm thick slab centered at the GB plane. The values of *μ* and frequency of GCMC insertions (*M/N*) change from case to case in our simulations to adjust the loading rate as well as the stoichiometry of inserted C/Si atoms. Loading a large amount of interstitials into a fixed volume supercell can lead to stress build up which in turn can affect subsequent GB evolution. To relax the accumulated stress, every 10 ps of MD simulations we turn off GCMC for 1 ps during which time we run MD simulations in the NPT ensemble. GCMC is then resumed with a fixed-volume supercell.

Ideally, one should control the interstitial loading rate of GCMC to be the same as the interstitial flux to GBs in SiC under irradiation. The interstitial flux predicted from *ab initio* based rate theory model^32^ is in the range of 10^11^ to 10^15^/m^2^∙s (see supplementary information for details). However, given the limited timescale of our simulation (up to hundreds of ns), it is impossible to simulate these relatively low fluxes. In order to identify GB structural evolution trend in such a short timescale, we run MD/GCMC with high interstitial loading rate (roughly 10^27^/m^2^∙s) at temperatures as high as 1500 K. Low temperature simulations such as 700 K and 1000 K are also conducted to check if the trend identified at 1500 K can be preserved. If a clear trend can be observed with high interstitial loading rate at all these temperatures within ns timescale, it is highly likely to happen under a much lower interstitial flux in experiments. It is also important to note that experimentally realistic loading rates are assumed in this paper when considering whether defects will accumulate at the GBs or diffuse along the GBs to other sinks.

## Electronic supplementary material

Supplementary Materials
