# Supplementary material for: Small-Angle Twist Grain Boundaries as Sinks for Point Defects
Source: Sci Rep. 2018 Feb 27;8:3736. doi: 10.1038/s41598-018-21433-7 (PMC5829279; doi:10.1038/s41598-018-21433-7)
Supplement: Supplementary file 1 — Supplementary Materials [file 41598_2018_21433_MOESM1_ESM.pdf]

Supplementary materials for

Small-Angle Twist Grain Boundaries as Sinks for Point Defects

Hao Jiang[1] (hjiang39@wisc.edu), Izabela Szlufarska[1,2](szlufarska@wisc.edu)

[1]Department of Materials Science and Engineering, [2]Department of Engineering Physics

University of Wisconsin-Madison, WI, 53706

1. Bi-crystal simulation cell and definition of twist angle

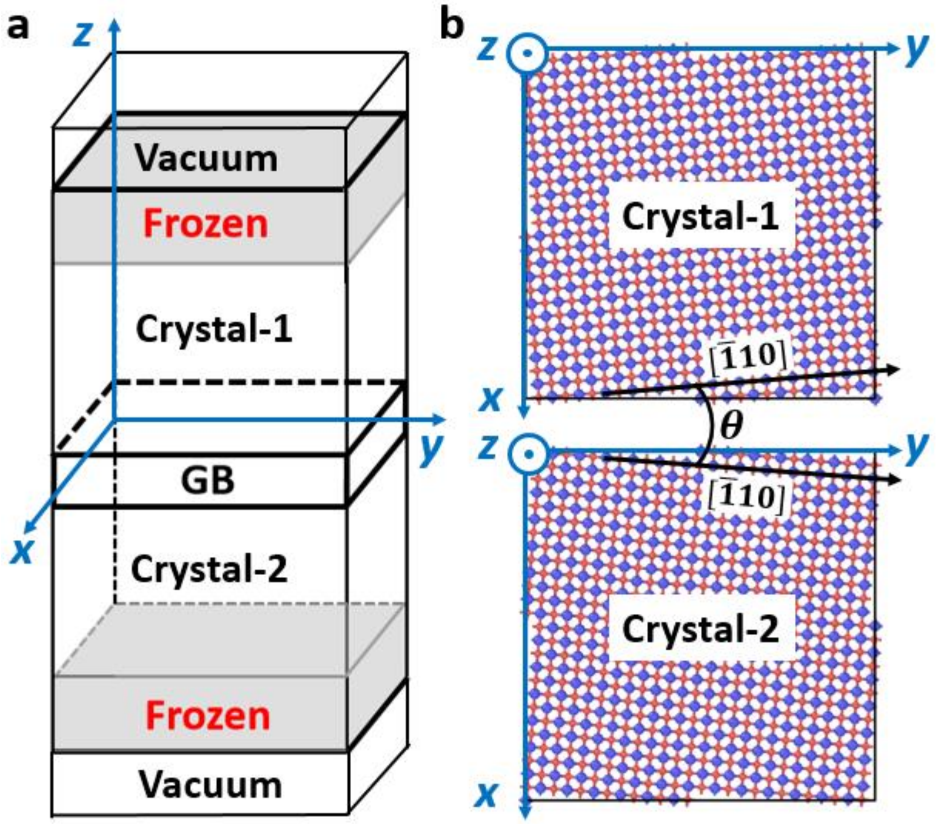

Figure S1 | **a**, bicrystal simulation cell; **b**, definition of twist angle using an example of (001) twist GB.

2. Types of {001} and {111} twist GBs investigated in this study

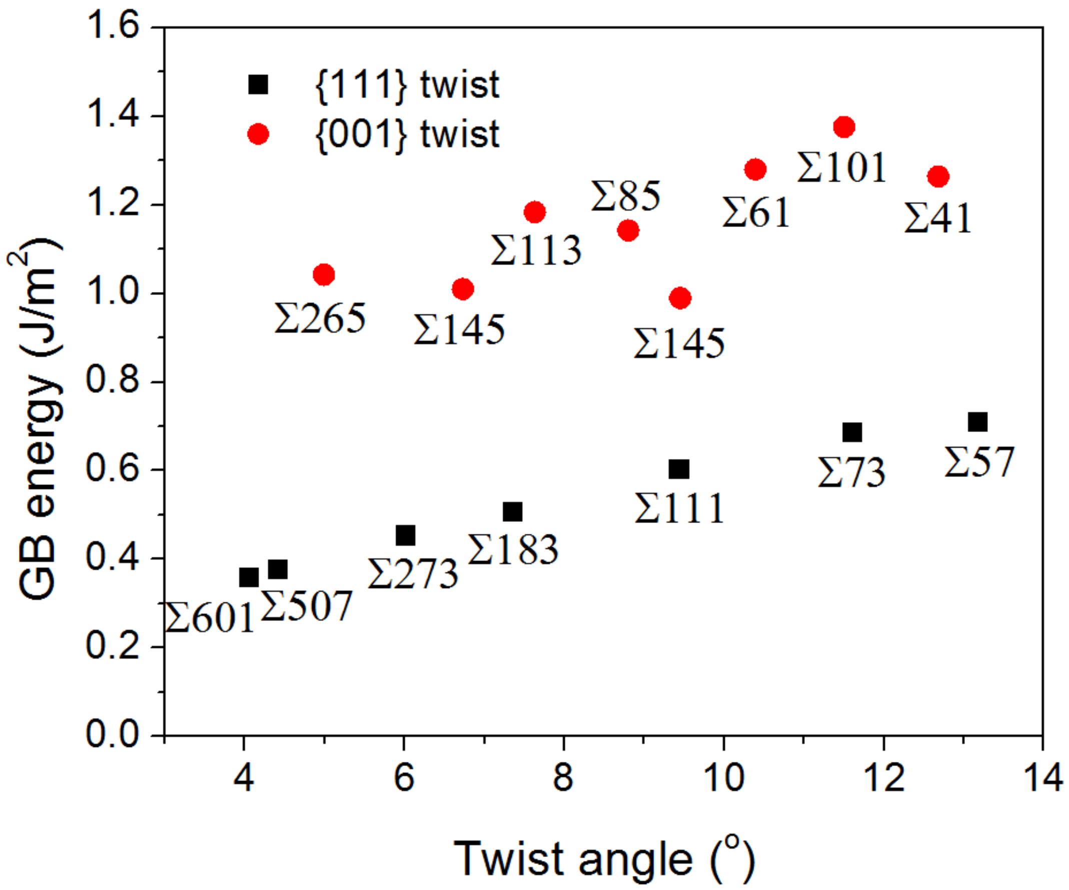

Figure S2 | Twist angles and GB energies of {001} and {111} twist GBs investigated in this study.

3. Analysis on defect kinetics in (001) Σ85 and (11$\bar{1}$) Σ507 twist GBs

Table S1 | List of irradiation conditions, grain sizes, interstitial fluxes to GBs predicted by rate theory model[1], and times of kinetic processes involved in GB evolution. t_{seg} is the average time interval between the arrivals of two successive interstitials from grain interior to the GB. t_{migr} is the average time for interstitial migration from crystalline-like region in a GB to dislocations in the GB. t_{sink} is the average time for interstitial migration along dislocation grid in GB to other sinks at the edges of a GB plane. Parameter *d* is the distance between dislocation intersections as defined in the main text. Green cells in t_{trap} correspond to the case when $t_{migr} << t_{seg}$. Yellow cells in t_{sink} are cases where $\mathrm{t}_{sink} >> t_{seg}$.

**Dose rate**	**Grain diameter**	**Temp.**	**Interstitial flux**	t_{seg}	**(001) ∑85 d = 2 nm**		**(111) ∑507 d = 3.8 nm**	
					t_{migr}	t_{sink}	t_{migr}	t_{sink}
(dpa/s)	(μm)	(K)	(#/m²s)	(s)	(s)	(s)	(s)	(s)
6.5×10^{-5}	10	873	6.7×10^{14}	1.5×10^{-5}	1.6×10^{-10}	1.9×10^{5}	3.0×10^{-10}	5.6×10^{1}
7.7×10^{-5}	10	873	5.4×10^{13}	1.8×10^{-4}	1.6×10^{-10}	1.9×10^{5}	3.0×10^{-10}	5.6×10^{1}
6.5×10^{-3}	0.1	573	6.0×10^{15}	1.7×10^{-2}	1.6×10^{-8}	1.2×10^{7}	3.2×10^{-8}	1.8×10^{5}
6.5×10^{-3}	1	750	5.0×10^{15}	2.0×10^{-4}	6.7×10^{-10}	1.2×10^{5}	1.3×10^{-9}	3.0×10^{2}
6.5×10^{-3}	0.1	573	1.1×10^{15}	9.2×10^{-2}	1.6×10^{-8}	1.2×10^{7}	3.2×10^{-8}	1.8×10^{5}
6.5×10^{-4}	0.1	573	3.5×10^{14}	2.9×10^{-1}	1.6×10^{-8}	1.2×10^{7}	3.2×10^{-8}	1.8×10^{5}
6.5×10^{-3}	1	873	2.0×10^{15}	5.0×10^{-4}	1.6×10^{-10}	1.9×10^{3}	3.0×10^{-10}	5.6×10
6.5×10^{-4}	1	873	9.3×10^{14}	1.1×10^{-3}	1.6×10^{-10}	1.9×10^{3}	3.0×10^{-10}	5.6×10
6.5×10^{-5}	10	1173	4.4×10^{13}	2.3×10^{-4}	1.6×10^{-11}	2.9×10^{2}	3.1×10^{-11}	1.2×10^{-1}
6.5×10^{-5}	1	873	3.0×10^{14}	3.4×10^{-3}	1.6×10^{-10}	1.9×10^{3}	3.0×10^{-10}	5.6×10
6.5×10^{-3}	1	1000	1.7×10^{14}	6.0×10^{-3}	5.0×10^{-11}	7.8×10^{1}	9.8×10^{-11}	2.7×10^{-2}
6.5×10^{-3}	1	1173	3.3×10^{13}	3.1×10^{-2}	1.6×10^{-11}	3.0×10	3.1×10^{-11}	1.2×10^{-2}
6.5×10^{-4}	1	1173	1.0×10^{13}	9.7×10^{-2}	1.6×10^{-11}	3.0×10	3.1×10^{-11}	1.2×10^{-2}
6.5×10^{-3}	0.1	873	6.4×10^{13}	1.6×10	1.6×10^{-10}	2.0×10^{1}	3.0×10^{-10}	5.7×10^{-1}
6.5×10^{-5}	0.1	873	6.4×10^{12}	1.6×10^{1}	1.6×10^{-10}	2.0×10^{1}	3.0×10^{-10}	5.7×10^{-1}
6.5×10^{-4}	0.05	873	9.7×10^{12}	4.1×10^{1}	1.6×10^{-10}	5.0×10	3.0×10^{-10}	3.0×10^{-1}
6.5×10^{-5}	0.05	873	3.1×10^{12}	1.3×10^{2}	1.6×10^{-10}	5.0×10	3.0×10^{-10}	3.0×10^{-1}
6.5×10^{-4}	0.1	1173	4.2×10^{12}	2.4×10^{1}	1.6×10^{-11}	3.1×10^{-2}	3.1×10^{-11}	1.2×10^{-3}
6.5×10^{-5}	0.1	1173	9.3×10^{11}	1.1×10^{2}	1.6×10^{-11}	3.1×10^{-2}	3.1×10^{-11}	1.2×10^{-3}

4. Simulations of interstitial loading onto (001) Σ85 twist GB at 1500K

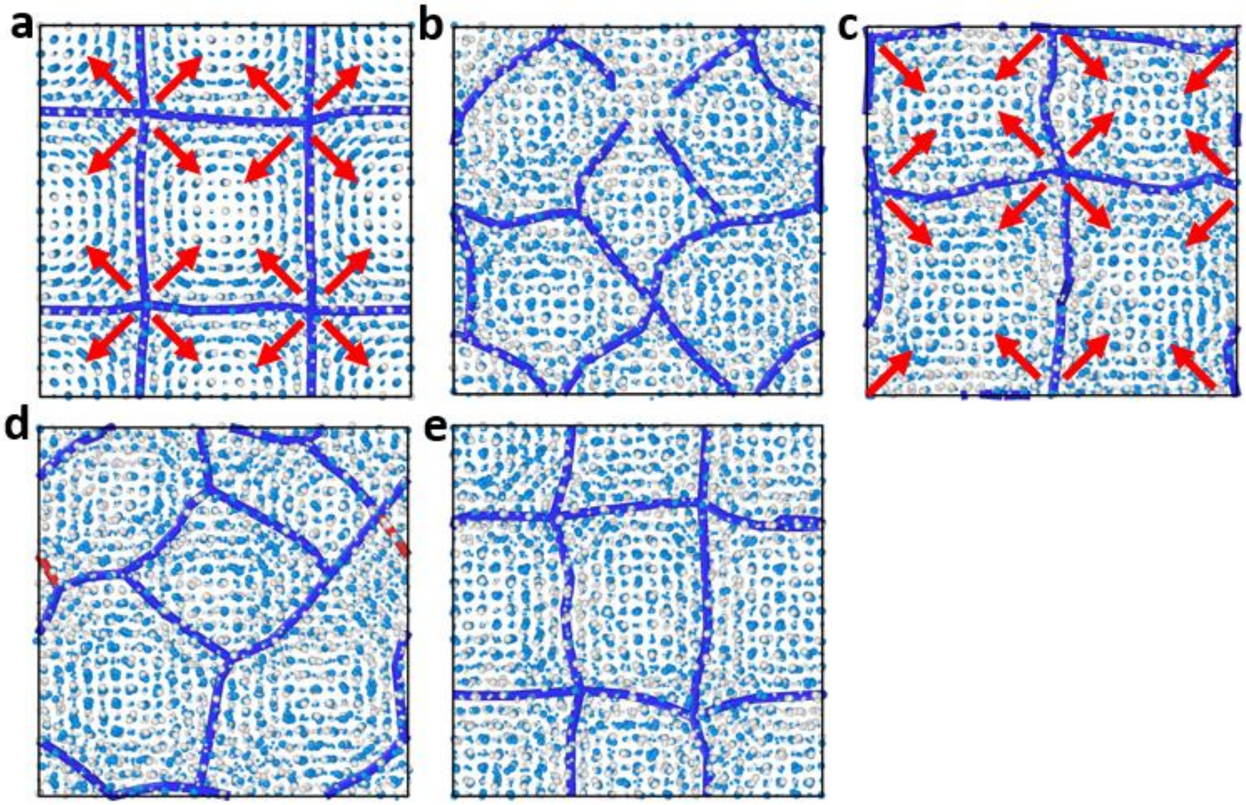

Figure S3 | Snapshots from simulations of interstitial loading with a C/Si stoichiometry of 1.3:1. GB structure **a**, before loading; **b**, loaded with 205 interstitials; **c**, loaded with 340 interstitials; **d**, loaded with 503 interstitials; **e**, loaded with 680 interstitials. The red arrows show the extension of interstitial loops from dislocation intersection. Large and small spheres represent Si and C atoms, respectively. Blue lines represent dislocations.

5. Energy landscape for C interstitial diffusion along partial dislocations at $(11\bar{1})$ Σ507 θ=4.4° twist GBs

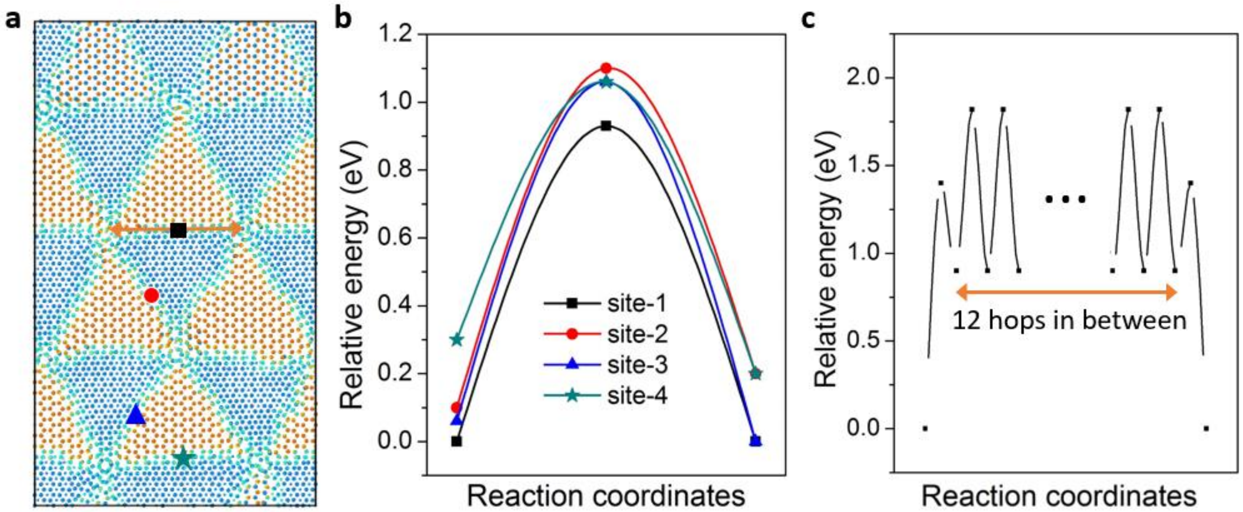

Figure S4 | a, Atomic structure of $(11\bar{1})$ Σ507 θ=4.4° twist GBs, blue spheres are atoms in crystalline regions, orange spheres are atoms in stacking faults, and green spheres are atoms on partial dislocations. The black square, red circle, blue triangle, and green star are sites where nudged elastic band calculations are conducted to sample C interstitial migration barrier. The orange arrow shows the diffusion path between two dislocation intersections; **b**, the migration barrier of C interstitials on 4 different sites in panel **a**; **c**, the approximated energy landscape for C interstitial diffusion between dislocation intersections. The averaged barrier height in panel **b** is used as an average migration barrier in panel **c** to approximate the energy landscape between the two intersections marked by orange line in panel **a**.

Bibliography

1. Swaminathan, N.; Morgan, D.; Szlufarska, I., Ab initio based rate theory model of radiation induced amorphization in β-SiC. *J. Nucl. Mater.* **2011,** *414* (3), 431-439.
